# Solid-State NMR Characterization of Mefloquine Resinate Complexes Designed for Taste-Masking Pediatric Formulations

**DOI:** 10.3390/ph17070870

**Published:** 2024-07-02

**Authors:** Leandro B. Borré, Eduardo G. R. Sousa, Rosane A. S. San Gil, Mateus M. Baptista, Alexandre A. Leitão, João M. A. R. De Almeida, Olívia Carr, Osvaldo N. Oliveira, Flávio M. Shimizu, Thiago F. Guimarães

**Affiliations:** 1 Instituto de Química, Universidade Federal do Rio de Janeiro, Rio de Janeiro 21941-909, RJ, Brazil; lbborre@iq.ufrj.br (L.B.B.); matemarinh@pos.iq.ufrj.br (M.M.B.); j.monnerat@iq.ufrj.br (J.M.A.R.D.A.); 2 Instituto de Tecnologia em Fármacos (Farmanguinhos), Fundação Oswaldo Cruz, Rio de Janeiro 21041-250, RJ, Brazil; eduardo.sousa@fiocruz.br (E.G.R.S.); thiago.frances@fiocruz.br (T.F.G.); 3 Instituto de Pesquisa de Produtos Naturais, Universidade Federal do Rio de Janeiro, CCS, Rio de Janeiro 21941-599, RJ, Brazil; 4 Departamento de Química, Universidade Federal de Juiz de Fora, Juiz de Fora 36036-900, MG, Brazil; alexandre.leitao@ufjf.edu.br; 5 São Carlos Institute of Physics, University of São Paulo, São Carlos 13566-590, SP, Brazil; oliviacarr@gmail.com (O.C.); chu@ifsc.usp.br (O.N.O.J.); 6 Department of Applied Physics, Institute of Physics (IFGW), University of Campinas (UNICAMP), “Gleb Wataghin”, Campinas 13083-970, SP, Brazil; fmshimizu@yahoo.com.br

**Keywords:** mefloquine, CPMAS NMR, ion-exchange resin, ^13^C NMR, T1*ρ*^H^ relaxation time, taste masking, electronic tongue

## Abstract

Mefloquine (MQ) is an antimalarial medication prescribed to treat or malaria prevention.. When taken by children, vomiting usually occurs, and new doses of medication frequently need to be taken. So, developing pediatric medicines using taste-masked antimalarial drug complexes is mandatory for the success of mefloquine administration. The hypothesis that binding mefloquine to an ion-exchange resin (R) could circumvent the drug’s bitter taste problem was proposed, and solid-state ^13^C cross-polarization magic angle spinning (CPMAS) NMR was able to follow MQ–R mixtures through chemical shift and relaxation measurements. The nature of MQ–R complex formation could then be determined. Impedimetric electronic tongue equipment also verified the resinate taste-masking efficiency in vitro. Variations in chemical shifts and structure dynamics measured by proton relaxation properties (e.g., T1*ρ*^H^) were used as probes to follow the extension of mixing and specific interactions that would be present in MQ–R. A significant decrease in T1*ρ*^H^ values was observed for MQ carbons in MQ–R complexes, compared to the ones in MQ (from 100–200 ms in MQ to 20–50 ms in an MQ–R complex). The results evidenced that the cationic resin interacts strongly with mefloquine molecules in the formulation of a 1:1 ratio complex. Thus, ^13^C CPMAS NMR allowed the confirmation of the presence of a binding between mefloquine and polacrilin in the MQ–R formulation studied.

## 1. Introduction

Mefloquine hydrochloride (MQ), when administered orally, either alone or in combination with artemisinin derivatives, is one of the primary agents used for the prevention, treatment, and emergency treatment of malaria infections caused by *Plasmodium vivax* and *Plasmodium falciparum* [1,2]. Other diseases can be treated with MQ. In a recent review, Dobhal et al. [3] examined the last five years of work using natural biomolecules (BMs) to counteract the ZIKA virus through virtual screening and in vitro investigations. In vitro testing has shown that mefloquine can reduce ZIKA virus infections in cell lines.

**Figure 1:**
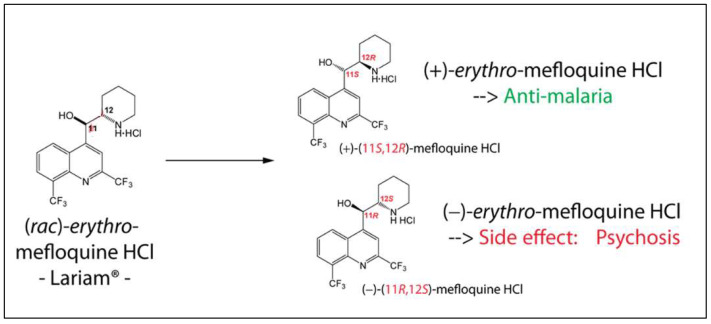
Structural formulas for racemic, (+), and (−) of mefloquine. HCl (Adapted from ref. [19]).

**Figure 2:**
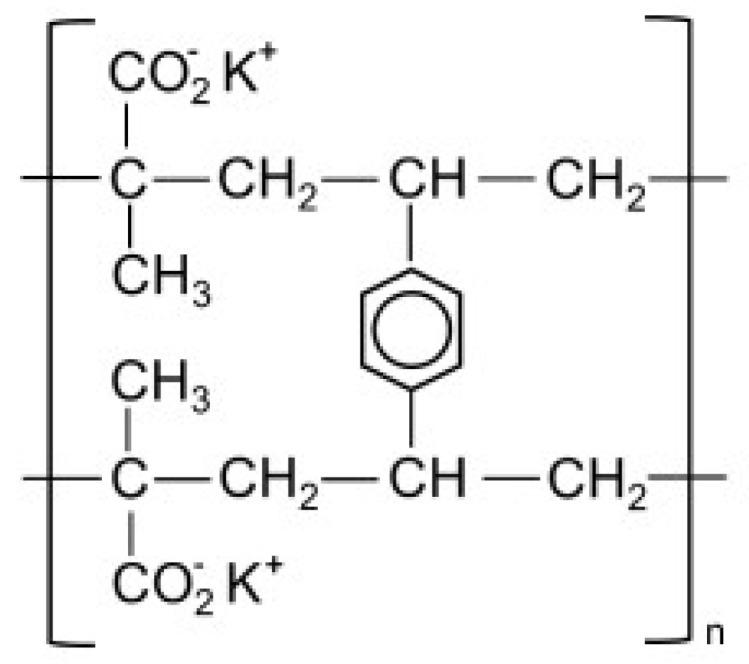
Structural formula of potassium polacrilin—‘2-Propenoic acid, 2-methyl-, potassium salt (1:1), polymer with diethenylbenzene’ (adapted from ref. [20]).

Due to the quinine moiety in its chemical structure (Figure 1), MQ has a highly unpleasant, bitter taste. The bitter taste of antimalarial drugs is pointed to as the most likely cause of non-compliance to treatment, mainly by children [4,5]. So, developing pediatric formulations that could mask the bitter taste of MQ would be a considerable advantage for the success of oral administration. However, adding flavors and sweeteners to the formulation may not be efficient enough to achieve acceptable drug palatability, requiring some technological process to improve the taste-masking efficiency [4,6]. Among the different techniques described in the literature to overcome taste-masking issues, such as microencapsulation [7] or formation of inclusion complexes [8], complexation with ion-exchange resins (IER) has received considerable attention. It has often proved to be an efficient method for masking the bitter taste of different drugs [9,10,11,12,13,14,15]. Guimaraes et al. [16] have investigated the taste masking of chloroquine–resin complexes using polacrilin potassium resin (Figure 2) and other ion-exchange resins. The results indicated increased taste properties of the chloroquine–polacrilin complex compared to chloroquine diphosphate. 

Mefloquine hydrochloride (MQ) is a weak base with a pKa of approximately 8.6, and solubility has been reported to range from very slightly soluble to slightly soluble in water at room temperature. According to IUPAC nomenclature, it is termed (R, S)-erythro-a-(2-piperidyl)-2,8-bis(trifluoromethyl)-4-chinolinmethanol-hydrochloride. The chemical structure is shown in Figure 1, with a molecular weight of 414.77 g/mol and presents a melting point of around 259–260 °C [17]. Polacrilin potassium resin is a weakly acidic cation-exchange resin widely used as a tablet disintegrant in oral dosage formulations of drug products [18].

Solid-state NMR spectroscopy is a powerful tool for organic and inorganic materials and crystalline or amorphous phases. Particularly for rare spins (as ^13^C, 1.1% natural abundance), direct observation is sometimes prohibitive, and indirect excitation by abundant spins (as ^1^H) allows for obtaining ^13^C NMR spectra in a reasonable time through a cross-polarization magic-angle spinning pulse sequence (CPMAS). It is known [21] that in a given phase, by using the CPMAS sequence, the dependence of the ^13^C magnetization, *S(t_c_)* on contact time t_c_, is given by Equation (1):

(1) $$S\left(t_{c}\right)=So\;\left(1-\exp(\frac{-t_{c}}{\;T_{CH}})\right)\exp(\frac{{-t}_{c}}{T_{1\rho }^{H}})$$

T_CH_ and T_1_*_ρ_*^H^ measurements could provide information on the possible interactions between distinct components in a mixture. The procedure has considered that separate domains may have different proton relaxation properties. As a result, differences in dynamics could be used to establish the extension of mixing and specific interactions that would be present between distinct domains. That approach has been applied successfully to synthetic [22] and natural polymer mixtures [23]. Molecular motion on the scale of several nanometers can be investigated by measuring the spin-lattice relaxation time of the ^1^H nucleus (T_1_*_ρ_*^H^) in a rotating system [24]. Recently, Durand et al. [25] addressed the interactions between mefloquine and cyclodextrins by ^1^H and ^19^F NMR in solution. They showed that the non-covalent interactions present enhanced the solubility of the inclusion complexes thus formed.

An electronic tongue is an analytical tool based on a multisensory system based on potentiometric, amperometric, voltammetric, or impedimetric transduction methods. Among them, the impedimetric method is attractive for non-faradaic processes and dismisses the use of reference and auxiliary electrodes. A “fingerprint” of the analyzed liquid is obtained from the electrical responses of an array of sensing units that provide a qualitative assessment of taste based on the global selectivity paradigm. Electronic tongues have been successfully applied for in vitro taste-masking assessment and will be applied to complement the NMR results [26,27].

In this work, ^13^C solid-state NMR with a cross-polarization magic angle spinning pulse sequence was used to probe the interactions in mefloquine–polacrilin through chemical shift variations and ^1^H relaxation times.

## 2. Results

### 2.1. Characterization of the Mefloquine Hydrochloride Sample (MQ) by Solid-State ^13^C NMR

^13^C CPMAS NMR spectra of MQ were acquired on a 9.4 T spectrometer under different acquisition conditions to obtain the NMR spectrum of solids under optimized conditions. The following parameters were varied: rotation speed (n_r_, Hz), recycle time (D1, s), and contact time (t_c_, μs).

#### 2.1.1. Rotation Speed Optimization

The rotation speed was optimized using our 4 mm probe, the maximum speed of which is 14 kHz. The speed was varied from 8000 to 12,000 Hz. Figure S1 (Supplementary Material) shows the spectra of the mefloquine hydrochloride sample, obtained at two speeds: 9800 and 10,000 Hz. The speed chosen was 9800 Hz, as it presented rotational bands that did not overlap with the isotropic signals of mefloquine hydrochloride. 

#### 2.1.2. Recycle Time Optimization

^13^C CPMAS NMR spectra with recycle time variation were acquired in a contact time of 1100 μs following the work of Guo et al. [28], varying the recycle time (D1) in the range of 3 to 20 s. The spectra obtained are shown in Figure S2 (Supplementary Material). The recycling time chosen was 20 s.

#### 2.1.3. Contact Time Optimization

**Figure 3:**
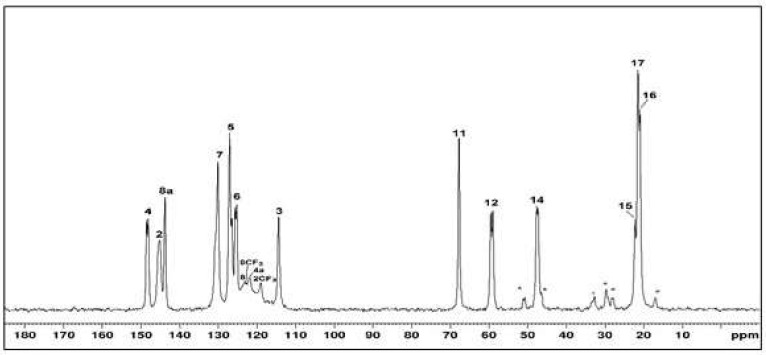
Optimized ^13^C CPMAS NMR spectrum obtained for MQ. Optimized conditions: NS-512, D1—20 s, contact time—10,000 μs. Asterisks denote spinning sidebands. The carbon signals are numbered following the MQ structure presented in Table 1.

The last optimized parameter was the contact time in the cross-polarization pulse sequence. The ^13^C CPMAS NMR spectra were obtained with a recycle time of 20 s, and the contact times were varied between 20 and 50,000 μs. Figure S3 (Supplementary Material) shows the results achieved. Under optimized conditions (Figure 3), the spectrum obtained in this work was comparable to that published by Guo et al. [28], which employed a 20 T spectrometer with an MAS frequency of 40,000 Hz. 

### 2.2. Characterization of the Ion-Exchange Resin Sample: Polacrilin Potassium (Sample R)

^13^C CPMAS NMR spectra of sample R were obtained at contact times in the 200 to 20,000 μs range, with a recycle time of 20 s. Figure S4 (Supplementary Material) shows the spectra obtained. The signals observed correspond to the CO_2_^−^K^+^ groups in the region between 190 and 180 ppm, to the aromatic ring carbons, around 150 ppm (C1) and in the region from 120 to 135 ppm (C2), and to the C, CH, CH_2_, and CH_3_ groups of the aliphatic chain of the resin from the methacrylate portion of the copolymer. 

### 2.3. Characterization of the Mefloquine Resinate Sample (MQ–R Sample)

**Figure 4:**
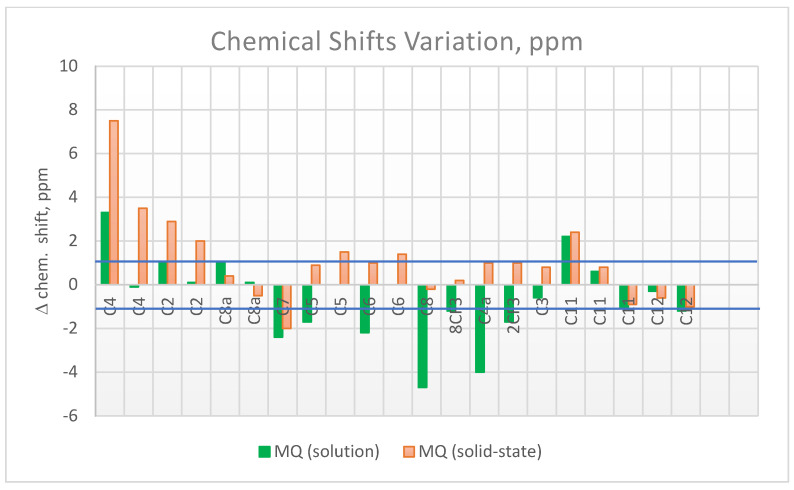
^13^C chemical shifts variation (D chemical shift = d_MQ–R_ − d_MQ_) observed for MQ carbon skeleton in DMSO-*d_6_* solution (green), and in the solid state (orange). The change for higher frequency (deshielding) is denoted by the positive bar; the shift for lower frequency (shielding) is denoted by the negative bar. DMQ chemical shifts (±1 ppm interval) for each carbon signal are indicated by the horizontal lines in blue.

The mefloquine–resin complex was prepared in line with previously published work [16]. ^13^C CPMAS NMR spectra of the mefloquine resinate sample were obtained under the same contact time conditions as the MQ sample (Figure S5, Supplementary Material). Significant changes in the chemical shifts of MQ in MQ–R were observed. Compared to what was observed in the pure MQ (Figure 3), some signals shifted for higher frequency (deshielding effect), and the unfolding of some signals was observed, as summarized in the bar graph of Figure 4. 

**Figure 5:**
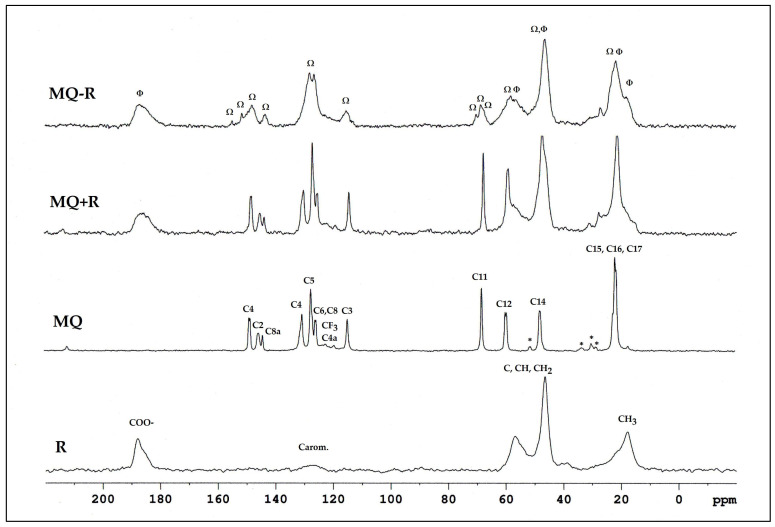
^13^C CPMAS NMR spectra of the samples: polacrilin resin (R, Φ); mefloquine hydrochloride (MQ, Ω); physical mixture MQ + R; and mefloquine–resin complex (MQ–R). * Denotes spinning sidebands.

Figure 5 shows the corresponding ^13^C CPMAS NMR spectra. A physical mixture of MQ and R (denoted MQ + R) was also prepared, and the ^13^C CPMAS spectrum was included for comparison. Sample MQ–R is distinct, presenting shifted and new signals, compared with samples MQ and R, whereas sample MQ + R showed the same signals as the samples MQ and R, but some were superposed. 

Some variations in chemical shifts, both in the carboxylate group of the resin and in the carbons present in the solid mefloquine structure, were also evident. The following can be highlighted:

- A broadening of the signal corresponding to the CO_2_^−^ groups: in R, it was observed at 186.5 and 185.6 ppm, and in MQ–R, between 187.7 and 181.6 ppm;
- In MQ, the signals of aromatic carbons, non-protonated C4 (unfolded in two, at 148.5 and 148.1 ppm), C2 linked to N and the CF_3_ group (at 145.2 ppm), and C8a of ring junction and linked to the N of the nitrogen ring (at 143.8 ppm), moved to 155, 150.6 ppm, 148.1, 147.2, and 143.3 ppm, respectively;
- In MQ, the signals from carbons C7, C5, and C6 of the benzene ring at 130.1, 127.1, and 125.2 ppm, respectively, shifted to a lower frequency, at 128 and 122.9 ppm in the MQ–R sample;
- The narrow signal from the protonated aromatic carbon C3, observed at 114.4 ppm in MQ, was observed as a broad signal at 115.2 ppm in the MQ–R sample.

The aliphatic carbon C11 (C-OH) signal was observed at 67.8 ppm in MQ; in MQ–R, it was split into three distinct, low-intensity signals at 70.2 ppm, 68.6 ppm, and 66.9 ppm. Table 1 lists the chemical shifts observed for the samples MQ, R, and MQ–R. Chemical shifts previously published in the literature for MQ in solid phase were also included for comparison, as were the ^13^C data of MQ in DMSO solution. The corresponding spectrum is depicted in Figure S6 (Supplementary Material).

### 2.4. Evaluation of Molecular Dynamics through T_CH_ and T_1ρ_^H^ Measurements

To investigate the interaction between mefloquine and polacrilin resin, a measure of T_CH_ (time constant of signal growth) and T_1_*_ρ_*^H^ (time constant of protons’ relaxation in the rotatory axis) by cross-polarization pulse sequence was performed. The bar diagram in Figure S7 (Supplementary Material) shows the T_CH_ variation obtained for MQ and R carbons. For the carbons of MQ, T_1_*_ρ_*^H^ relaxation times are in the range of 100–200 μs (Table 2). In contrast, for the carbons of polacrilin resin (R), T_1_*_ρ_*^H^ relaxation times showed a shorter value, between 9 and 11.7 ms (Table 3), thus evidencing that the carbons in polacrilin are rigid in the structure lattice. Conversely, the T_CH_ values depend primarily on the carbon type: in MQ, it varies from 300 to 500 μs for CH_2_ and CH sp^3^ carbons of the piperazine ring and >700 μs for aromatic and pyridine CH and C. In sample R, the T_CH_ values were between 99 and 640 μs.

**Figure 6:**
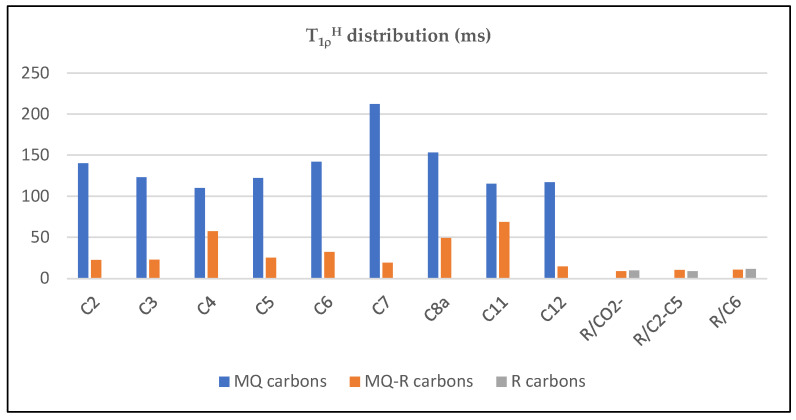
Distribution of T_1_*_ρ_*^H^ for the carbons of mefloquine hydrochloride, MQ (blue); MQ–R (orange); and resin R (grey).

**Figure 7:**
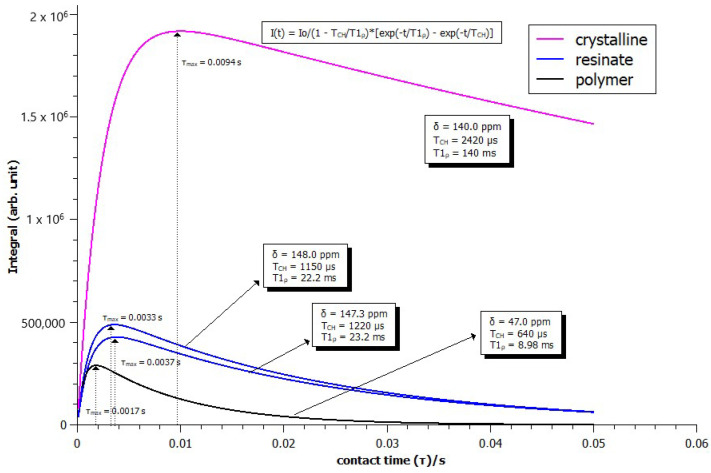
Variation of the intensity of carbon C2 signal with the cross-polarization contact time (tc, s) for samples of mefloquine hydrochloride (MQ, pink) and mefloquine resinate (MQ–R, blue). The curve obtained for polacrilin carbons at 47 ppm (R, black) was included for comparison. The vertical scale is arbitrary.

The bar diagram depicted in Figure 6 illustrates the results of molecular dynamics probed by T_1_*_ρ_*^H^ relaxation values, listed in Table 2 and Table 3 for each carbon of MQ, MQ–R, and R. Sample MQ–R presented T_1_*_ρ_*^H^ relaxation times for carbons in the range of 14.8 to 68.4 μs, highly contrasting with the values measured for MQ. On the other hand, those values are in the same order of magnitude as polacrilin resin R. Figure 7 depicts the curves of intensity variation with contact times obtained for carbon C2 in MQ USP and MQ–R samples. Figures S8–S12 (Supplementary Material) illustrate the dependence of C signal intensity on the contact time for carbons C2, C7, C5, C6, and C12 in MQ and MQ–R samples, respectively. The curves obtained for the carbons of sample R are shown in Figure S13 (Supplementary Material).

### 2.5. In Vitro Taste-Masking Evaluation

**Figure 8:**
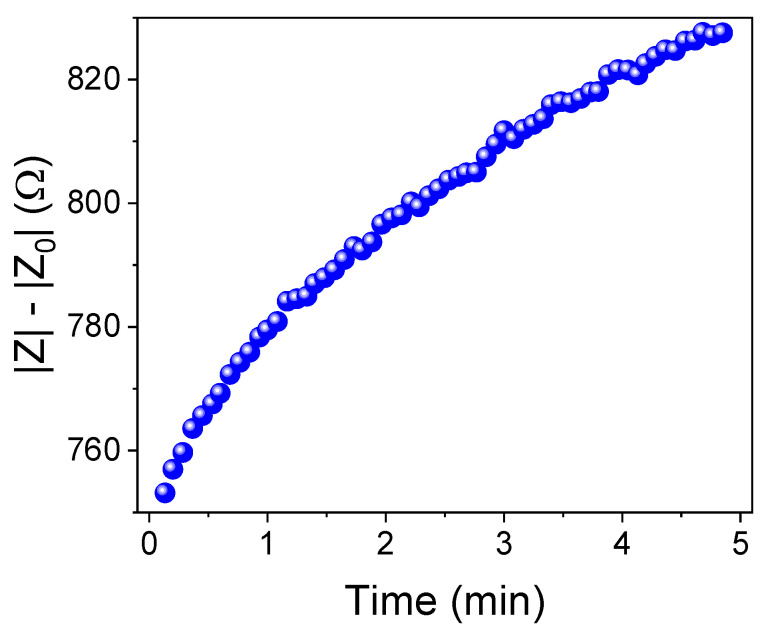
The dissolution test for mefloquine resinate in simulated salivary conditions was performed using the impedance method, measured through the conductivity difference between MQ–R (|Z|) and R (|Z_0_|).

Electrical impedance measurements were performed at 1 kHz for 5 min in the simulated salivary solution. Considering the difference between the response of the resin in the presence (Z) of MQ and the absence of the mefloquine drug (Z_0_), the drug release kinetic was monitored (Figure 8). A theoretical exponential fitting of the experimental data suggests that most of the drug was released from the MQ–R sample within 3.4 min. Such liberation decreased the solution conductivity from ~753 to 827 Ω, with a dispersion of ~5% for two replicates.

**Figure 9:**
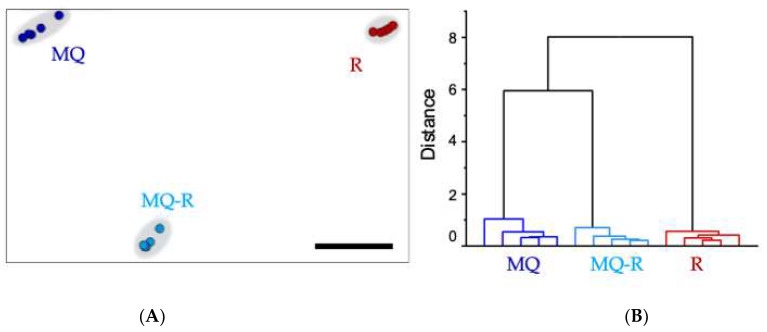
The taste-masking efficiency was determined with an electronic tongue analysis. (**A**) IDMAP plot points to the clusters formed by the mefloquine (MQ), the resin (R), and the mefloquine resinate (MQ–R). (**B**) Dendrogram with the Euclidean distances and similarities between the clusters formed.

An impedimetric electronic tongue was also used to assess the taste-masking effectiveness and confirm the dissolving results. The magnitude impedance spectra of mefloquine (MQ), polacrilin potassium resin (R), and the mefloquine resinate (MQ–R) were projected using interactive document mapping (IDMAP) and hierarchical cluster analysis (HCA) pattern recognition methods [16]. The assay was carried out using an array of six sensing units. The IDMAP plot, Figure 9A, shows a clear distinction of the three solutions, which is the reason for achieving a high silhouette coefficient value of 0.96 (from 0.71 to 1, a high data correlation is obtained). The drug and the resin clusters are displaced on the opposite edges of the map. Meanwhile, the MQ–R complex is in the center. However, the distance between MQ and MQ–R is smaller than between MQ–R and R, which suggests that the bitter taste of mefloquine was not completely masked. The Euclidean distances between the clusters are projected in Figure 9B through the dendrogram, confirming the last result.

## 3. Discussion

Mefloquine (MQ) is an essential drug that requires a slightly acidic medium to ionize its salt. This ionization allows the drug to complex with the ion-exchange resin polacrilin potassium and form mefloquine resinate (MQ–R). At pH 5.0, the resin displayed a Drug Loading Efficiency of 99.64 ± 0.14%, expressed as mefloquine hydrochloride. The pKa range of ion-exchange resins with carboxylic acid groups is between 4.0 and 6.0 [29]. This range is also where MQ is ionized (pKa 8.6), facilitating the formation of resinate complexes.

^13^C CPMAS NMR spectra of the MQ were acquired under different acquisition conditions to obtain the NMR spectrum of solids under optimized conditions. The following parameters were varied: rotation speed (nr, Hz), recycle time (D1, s), and contact time (tc, μs). Solution ^13^C NMR spectrum (DMSO-*d_6_*, a polar aprotic solvent) was also acquired for comparison. Prado et al. [1] employed a rotation speed of 15 kHz to obtain the ^13^C CPMAS NMR spectrum of mefloquine, and Guo et al. [28] used probes that allowed the sample to be rotated between 20 and 60 kHz. Previous experiments indicate that the optimized spin rate for MQ was 9500 to 10,500 Hz. The speed chosen was 9800 Hz, as it presented rotational bands that did not overlap with the isotropic signals of mefloquine hydrochloride. It is worth mentioning that the spectrum obtained was comparable to that published by Guo et al. [28]. ^13^C CPMAS NMR spectra with recycle time variation were acquired in a contact time of 1100 μs following the work of Guo et al. [28], varying the recycle time (D1) from 3 to 20 s. Although a recycle time of 5 s was used in the literature, more resolved signals were obtained with 20 s, so this value was chosen in this work.

The last optimized parameter was the contact time in the cross-polarization pulse sequence. The ^13^C CPMAS NMR spectra were obtained with a recycle time of 20 s, and the contact times were varied between 20 and 50,000 μs. Prado et al. [1] obtained the spectrum of mefloquine hydrochloride with a contact time of 1500 μs; the authors did not visualize the signals corresponding to the CF_3_ carbons of the mefloquine structure in the expected position in the highest frequency region of the spectrum. Guo et al. [28] published data on mefloquine’s solid-state ^13^C NMR spectra, with a rotation speed of 20 kHz and contact times between 0.5 and 1.4 μs. Under optimized conditions (Figure 3), the spectrum obtained in this work was comparable to that published by Guo et al. [28]. It was found that the signals from carbons linked to fluorine, in the region between 115 and 125 ppm, could be identified in the spectrum obtained with a longer contact time. Also, in contrast with the published spectrum, the carbon signals of the piperidine ring (C15, C16, and C17) could be seen with ν_r_ 9800 Hz, D1 20 s, and t_c_ 10,000 μs.

Polacrilin potassium is the salt of a crosslinked polymer derived from methacrylic acid, resulting in a weakly acidic form of a cationic ion-exchange resin. The fact that the spectrum with the highest intensity signals was observed in a shorter contact time range (Figure S4) confirms the rigid structure of the polymeric resin analyzed. The spectra of MQ–R showed significant changes in the chemical shifts in comparison with MQ sample spectra. As can be seen in Table 1, the presence of more than one signal for carbons C4, C5, C6, C12, and C14 in the ^13^C CPMAS NMR spectrum of MQ suggests that in the sample analyzed, there is a mixture of diastereoisomers since, in the case of this compound, only one crystalline structure is reported [28]. In the case of C12 and C14 carbons, the presence of more than one signal would also result from dipolar interaction with the quadrupolar ^14^N neighbor. Conversely, in the spectrum of MQ–R, the carbons C4, C2, C8a, and C11 were unfolded in two or three signals. That result would indicate that the diastereoisomers interact with the resin in distinct forms that can be detected in the solid-state ^13^C spectrum. Comparing the chemical shifts of MQ in solution with the ones obtained for solid MQ and MQ–R complexes, it could be observed that the packing effect in MQ solid vanishes in the sample MQ–R: the chemical shifts of carbons C8, C4a, C7, and C6 in MQ–R are somewhat more shielded to the ones in MQ in DMSO solution (Figure 4, green bars). From that result, we can suggest that in a 1:1 MQ–R complex, the MQ is highly dispersed in the R moiety through an electron donation mechanism from resin to MQ structure that shields the trifluoromethyl benzenoid ring. The presence of R also affected MQ’s molecular dynamics. In the pure MQ, an increase in contact time caused an increase in signal intensities until 10,000 contact time. Still, in contrast, in the case of MQ–R, it was possible to observe a decrease in the signal intensities of the MQ carbons with the increased contact time, with molecular dynamics like those presented by pure resin. Distinct molecular dynamics were established between MQ and resin by observing the curves of variation of intensity of signals in the function of contact time t_c_ (Figures S8–S12, Supplementary Material). For MQ, the maximum is reached at around 10,000 μs, whereas for polacrilin resin, this value is about 1000 μs, except for COO^-^ carbon (Figure S13, Supplementary Material). Strongly dipolar-coupled hydrogen nuclei in rigid solids tend to have similar T_1_*_ρ_*^H^ values because spin diffusion is fast on the NMR timescale [21]. However, T_CH_ is determined by the local strength of the dipolar coupling between C and H and is specific to the C environment. We observed that for the non-protonated C2 and C8a of MQ, the T_CH_ values were higher than the protonated sp^2^ carbons. On the other hand, following the theory, the sp^3^ carbons presented the lowest T_CH_ in the 300 to 500 s range. T_CH_ values decreased for all the carbons measured for the MQ–R complex except for carbon C5 (Table 2). The most prominent decrease was observed for CH sp^3^ carbons C11 and C12. That result suggests that MQ in MQ–R strongly interacts with the resin structure through a proton spin diffusion mechanism. Durand et al. [25] showed that the non-covalent interactions between mefloquine and cyclodextrins enhanced the solubility of the inclusion complexes thus formed in solution. Further studies will be conducted to establish the structure of the MQ–R complex.

The electronic tongue system evaluated the taste-masking efficiency of the MQ, R (resin), and resinate (MQ–R). The impedance spectra of all three samples were analyzed using IDMAP and HCA pattern recognition techniques. The IDMAP method further converted the dissimilarities between each spectrum to Euclidean distance and discriminated the clusters formed by resinate (MQ–R) from those of MQ. The distance between the cluster of resinate and that of the drug increased with the effectiveness of the taste masking. The results pointed out that the bitter taste of MQ was not wholly masked, but it was sufficiently compelling to make MQ tolerable for pediatric patients. This method has been considered effective in evaluating drug taste masking [16,30] and can be applied in the early stages of product development.

## 4. Materials and Methods

### 4.1. Materials

Mefloquine hydrochloride (MQ, C_17_H_16_F_6_N_2_O.HCl) was supplied by Farmanguinhos/Fiocruz, Rio de Janeiro, RJ, Brazil. Mefloquine–resin complexes (MQ–Rs) were prepared in a 1:1 drug-to-resin ratio using polacrilin potassium (R, Amberlite^TM^ IRP 88) from DuPont Water Solutions, Edina, MN, USA, and kindly provided by Colorcon do Brasil, Cotia, SP, Brazil. 

### 4.2. Mefloquine Resinate Preparation

Equal amounts of MQ and resin (drug: resin ratio 1:1 *w*:*w*) were weighed and suspended in an HCl aqueous solution (pH 5.0) and kept under magnetic stirring for 24 h at room temperature. Resinates were separated by vacuum filtration and dried in a vacuum oven Memmert VO 200 (Memmert GmbH + Co.KG, Schwabach, Germany) at 60 °C and 1100 mbar for 48 h. The supernatant was filtered through a 0.45-μm membrane filter and measured by UV-visible spectrophotometry at λ = 222 nm [31]. The drug loading efficiency (%) was calculated by comparing the amount of the drug before and after complex formation, according to Equation (2).

(2) $$Drug\;loading\;efficiency\;\left(\%\right)=\frac{mass\;of\;MQ-mass\;of\;MQ\;unbounded}{mass\;of\;MQ}\times 100$$

### 4.3. Solid-State NMR Analyses

#### 4.3.1. Sample Preparation

The samples, as received, were transferred to zirconium oxide rotors (4 mm diameter). After weighing, the rotors were closed using 4 mm Kel-F caps.

#### 4.3.2. Acquisition Conditions for ^13^C Solid NMR Spectra

^13^C Solid-state NMR spectra were acquired using Bruker equipment, model Avance III WB400, 9.4 T, equipped with a 4 mm triple channel probe. The spectra acquisition conditions were as follows: pulse sequence—cross-polarization magic angle spinning; spinning rate—between 8 and 12 kHz; recycle time—between 3 and 20 s; contact time for cross-polarization—between 0.02 and 50 ms. During the signal acquisition step, a two-pulse phase-modulated (TPPM) sequence was used for proton decoupling [32]. Glycine was used as a reference (C=O 176.03 ppm).

#### 4.3.3. Measurement of T_CH_ and T_1_*_ρ_*^H^ Relaxation Times

The relaxation time values were measured indirectly through the cross-polarization sequence. To this end, ^13^C CPMAS NMR spectra were acquired, with variation in contact times ranging from 20 μs to 50,000 μs, rotation speed of 9800 Hz, and recycle time of 20 s. Bruker’s Dynamics Center data processing platform version 2.8.3 (12 January 2023) was used to obtain the signal growth time constants and the decay time constant of the signals on the rotational axis for the different signals.

Relaxation time values for all carbons were possible for the mefloquine hydrochloride sample. The same happened for the resin. In the case of the mefloquine resinate sample, the overlap of some MEF signals with resin signals restricted the range of measurements.

### 4.4. Taste-Masking Evaluation

#### Impedimetric Electronic Tongue

An impedimetric electronic tongue system performed an in vitro taste-masking assessment [33]. The dissolution assay was performed in artificial salivary fluid. Here, an array of six sensing units were built by modifying five interdigitated gold electrodes with five bilayers of LbL film (layer-by-layer) of poly(ethyleneimine) (PEI), poly(styrene sulfonate) (PSS), poly(3,4-ethylene dioxythiophene)/poly(styrene sulfonate) (PEDOT/PSS), poly(pyrrole) (PPy), poly(aniline) (PANI), chitosan extracted from shrimp (Chit), and tetrasulfonated nickel (II) phthalocyanine (NiTsPc), obtaining the following architectures: (PEI/NiTsPc)_5_, (PANI/PSS)_5_, (PEI/PEDOT/PSS)_5_, (PEI/PPy)_5_, and (Chit/PSS)_5_. All the aqueous solutions were prepared with ultrapure water (Millipore Direct-Q5 system) at 0.5 g·L^−1^ at pH = 3 (adjusted with HCl solution). The electrical impedance spectroscopy assay was performed in the 1–1 × 10^6^ Hz frequency range with an AC voltage of 25 mV at room temperature. The pattern recognition methods used in this work were interactive document mapping (IDMAP) and hierarchical cluster analysis (HCA) [34].

## 5. Conclusions

The ^13^C CPMAS NMR spectra under different acquisition conditions allow us to characterize the samples of mefloquine hydrochloride, polacrilin resin, and mefloquine resinate. The comparison of the T_1_*ρ*^H^ values for the carbons of mefloquine, ion-exchange resin, and a mefloquine–resin complex allows us to confirm the absence of a physical mixture and the presence of the complex in a formulated sample of mefloquine–resin. Furthermore, the electronic tongue is a viable economical alternative for drug development screening. The results show that the API mefloquine could be distinguished from complex resinate and ion-exchange resin, proving that the taste masking was effective. Although it did not completely cover the API, it was sufficiently compelling to make MQ tolerable for pediatric patients. The mefloquine resinate complex presents itself as a feasible option for potential pediatric formulations in the future. The incorporation of excipients such as sweeteners and flavorings into the ultimate formulation will notably contribute to masking the inherently bitter taste of mefloquine.

## Figures and Tables

**Table 1:** Chemical shifts (δ, ppm) observed for the samples mefloquine hydrochloride (MQ), mefloquine resinate (MQ–R), and polacrilin potassium resin (R).

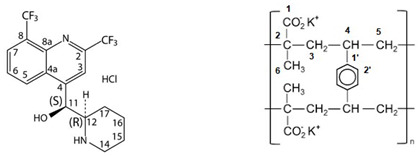						
Carbon Assignment	Mefloquine Hydrochloride (MQ)				Polacrilin (R)	Mefloquine Resinate (MQ–R)
	δ, ppm ^a^	δ, ppm ^b^	δ, ppm ^c^	δ, ppm ^d^	δ, ppm	δ, ppm
C4	151.71	148.5; 148.1	147.5	140–160	-	155.0 ^d^; 151.6 ^e^
C2	147.13	145.2	144.3	140–160	-	148.1 ^d^; 147.2 ^e^
C8a (C9) ^d^	143.22	143.8	143.6	130	-	144.2 ^d^; 143.3 ^e^
C7	130.39	130.1	129.7	130	-	128.0 ^e^
C5	129.72	127.1; 126.5	126.8	130	-	128.0 ^e^
C6	128.83	125.6; 125.2	125.2	130	-	126.6 ^e^
C8	127.57	123.1	122.2	110	-	122.9 ^e^
8-CF_3_	124.12	122.7	123.0	17; 9	-	122.9 ^e^
C4a (C10) ^d^	126.85	121.9	120.8	140–160	-	122.9 ^e^
2-CF_3_	121.65	119.0	120.1	17; 9	-	120.0 ^e^
C3	115.84	114.4	114.3	110	-	115.2
C11	68.01	67.8	67.7	70	-	70.2; 68.6; 66.9
C12	59.19	59.5; 59.0	59.3	60	-	58.9; 58.0
C14	44.69	47.7; 47.3	47.0	45	-	Overlap R
C15	22.02	22.3	23.1	20	-	Overlap R
C16	21.27	21.0	21.0	20	-	Overlap R
C17	21.56	21.5	21.7	20	-	Overlap R
C1 (CO_2_^−^K^+^)	-	-	-	-	186.5; 185.6	187.7; 184.8; 181.6
C1′arom.	-	-	-	-	148.4	Overlap MQ
C2′arom.	-	-	-	-	129.6; 127.8	Overlap MQ
C2, C3, C4, C5	-	-	-	-	~57	~57
C2, C3, C4, C5	-	-	-	-	~47	~46
C6	-	-	-	-	~20	~18

^a^ solution spectrum (DMSO), this work; ^b^ this work; ^c^ ref. [23]; ^d^ ref. [1]; ^e^ assignment suggested.

**Table 2:** Chemical shifts (δ, ppm) and T_CH_ and T_1_*_ρ_*^H^ relaxation times obtained for mefloquine hydrochloride (MQ) and mefloquine resinate (MQ–R).

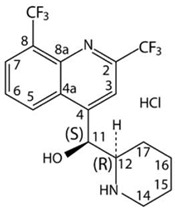						
Mefloquine	δ, ppm		T_CH_, μs		T_1_*_ρ_*^H^, ms	
Carbon	MQ	MQ–R	MQ	MQ–R	MQ	MQ–R
C4	148.5	154.9 *	978 ± 70.7	821 ± 417	114 ± 17.2	n.d.
	148.1	151.6 *	1000 ± 63.3	705 ± 236	110 ± 14.4	57.3 ± 23.8
C2	145.2	148.0 *	2420 ± 177.3	1150 ± 228	140 ± 27.6	22.2 ± 4.6
	-	147.3 *	-	1220 ± 268	-	23.2 ± 5.3
C8a	143.8	144.1 *	6380 ± 297.1	2410 ± 616	153 ± 22.5	49.1 ± 17.1
	-	143.6 *	-	2620 ± 522	-	35.6 ± 8.5
C7	130.1	128.8 *	1950 ± 294	695 ± 187	212 ± 119	19.1 ± 3.9
C5	127.1	128.0 *	689 ± 83.8	675 ± 260	122 ± 25.8	25.1 ± 6.0
	126.5	127.5	725 ± 84.1	614 ± 179	119 ± 24.5	21.6 ± 4.4
C6	125.6	126.6 *	1620 ± 199.5	746 ± 246	142 ± 46.7	32.3 ± 7.7
	125.2	126.0	n.d.	564 ± 258	n.d.	27.9 ± 11.0
C8	123.1	122.9 *	n.d.	n.d.	n.d.	n.d.
8-CF_3_	122.7	122.9 *	n.d.	n.d.	n.d.	n.d.
C4a	121.9	122.9 *	n.d.	n.d.	n.d.	n.d.
2-CF_3_	119.0	120.0 *	n.d.	n.d.	n.d.	n.d.
C3	114.4	115.3	737 ± 104	532	123 ± 35.9	22.5 ± 10.7
	-	114.7	-	n.d.	-	n.d.
C11	67.8	70.2	440 ± 77.8	67.8 ± 39.7	115 ± 16.2	68.4 ± 28.4
	-	68.6	-	n.d.	-	n.d.
C12	59.5	58.1	310 ± 44.3	68.1 ± 30.8	117 ± 18.9	14.8 ± 3.2
	59.0	overlap/R	406 ± 50.2	n.d.	111 ± 13.7	n.d.
C14	47.7	overlap/R	457 ± 68.1	n.d.	123 ± 22.8	n.d.
	47.3	overlap/R	461 ± 74.3	n.d.	121 ± 17.4	n.d.
C15	22.3	overlap/R	302 ± 88.6	n.d.	124 ± 24.1	n.d.
C17	21.5	overlap/R	433 ± 49.3	n.d.	117 ± 15	n.d.
C16	21.0	overlap/R	438 ± 44.7	n.d.	118 ± 13.8	n.d.

n.d.—not determined. * assignment suggested.

**Table 3:** Chemical shifts (δ, ppm), T_CH_, and T_1_*_ρ_*^H^ relaxation times obtained for carbons of polacrilin potassium resin in sample R and a mefloquine resinate (MQ–R) sample.

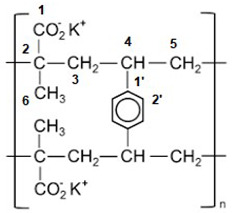						
Resin	δ, ppm		T_CH_, μs		T_1_*_ρ_*^H^, ms	
Carbons	R	MQ–R	R	MQ–R	R	MQ–R
C1 (CO_2_^−^K^+^)	186.5	187.3	1930 ± 387	1190 ± 200	9.7 ± 1.9	8.9 ± 1.4
C1′arom.	148.4	Overlap MQ	n.d.	n.d.	n.d.	n.d.
C2′arom.	129.6	Overlap MQ	n.d.	n.d.	n.d.	n.d.
	127.8	Overlap MQ	n.d.	n.d.	n.d.	n.d.
C2, C3, C4, C5	~57	~57; overlap MQ	99 ± 31	68 ± 31	9.4 ± 1.1	14.8 ± 3.2
C2, C3, C4, C5	~47	~46	640 ± 103	281 ± 85	9.0 ± 1.1	10.5 ± 1.9
C6	~20	~18	355 ± 107	280 ± 87	11.7 ± 2.5	9.3 ± 1.3

n.d.—not determined.

## Data Availability

Data are contained within the article and Supplementary Materials.

## Supplementary Materials

The following supporting information can be downloaded at https://www.mdpi.com/article/10.3390/ph17070870/s1, Figure S1: ^13^C CPMAS NMR spectra of mefloquine hydrochloride obtained at 9800 and 10000 Hz spinning frequencies.; Figure S2: ^13^C CPMAS NMR spectra of mefloquine hydrochloride (sample MQ), with contact time 1100 μs and variation of recycle times (s); Figure S3: ^13^C CPMAS NMR spectra of mefloquine hydrochloride (sample MQ), with a variation in contact time (in μs); Figure S4: ^13^C CPMAS NMR spectra of potassium polacrilin resin (sample R), obtained with a variation in contact time (in μs); Figure S5: ^13^C CPMAS NMR spectrum of the mefloquine resinate (sample MQ–R), with a variation in contact time (in μs); Figure S6: ^13^C solution NMR spectrum (*DMSO*-*d_6_*) of sample MQ; Figure S7: Distribution of T_CH_ for the carbons of mefloquine, MQ (blue), MQ–R complex (orange), and resin R (green); Figure S8: Dependence of C2 signal intensity (vertical scale, arbitrary unities) on the contact time t_c_ (horizontal scale, in s unities) for the samples mefloquine hydrochloride (MQ) and mefloquine–polacrilin complex (MQ–R); Figure S9: Dependence of C7 signal intensity (vertical scale, arbitrary unities) on the contact time t_c_ (horizontal scale, in s unities) for the samples mefloquine hydrochloride (MQ) and mefloquine–polacrilin complex (MQ–R); Figure S10: Dependence of C5 signal intensity (vertical scale, arbitrary unities) on the contact time t_c_ (horizontal scale, in s unities) for the samples mefloquine hydrochloride (MQ) and mefloquine–polacrilin complex (MQ–R); Figure S11: Dependence of C6 signal intensity (vertical scale, arbitrary unities) on the contact time t_c_ (horizontal scale, in s unities) for the samples mefloquine hydrochloride (MQ) and mefloquine–polacrilin complex (MQ–R); Figure S12: Dependence of C12 signal intensity (vertical scale, arbitrary unities) on the contact time t_c_ (horizontal scale, in s unities) for the samples mefloquine hydrochloride (MQ) and mefloquine–polacrilin complex (MQ–R); Figure S13: Dependence of C signal intensity (vertical scale, arbitrary unities) on the contact time t_c_ (horizontal scale, in s unities) for carbons in the sample of polacrilin resin (R).
