# Supplementary material for: Solid-State NMR Characterization of Mefloquine Resinate Complexes Designed for Taste-Masking Pediatric Formulations
Source: Pharmaceuticals (Basel). 2024 Jul 2;17(7):870. doi: 10.3390/ph17070870 (PMC11280060; doi:10.3390/ph17070870)
Supplement: Supplementary file 1 [file pharmaceuticals-17-00870-s001.zip › pharmaceuticals-3060464-supplementary.pdf]

Supplementary Material

Solid-State NMR Characterization of Mefloquine Resinate Complexes Designed for Taste- Masking Pediatric Formulations

Leandro B. Borré [1], Eduardo G. R. Sousa [2], Rosane A. S. San Gil [1,3,*], Mateus M. Baptista [1], Alexandre A. Leitão [4], João M. A. R. De Almeida [1], Olívia Carr [5], Osvaldo N. Oliveira Jr. [5], Flávio M. Shimizu [6] and Thiago F. Guimarães [2]

[1] Universidade Federal do Rio de Janeiro, Instituto de Química, Rio de Janeiro – RJ, Brazil; lbborre@iq.ufrj.br; matemarinh@pos.iq.ufrj.br; j.monnerat@iq.ufrj.br
[2] Fundação Oswaldo Cruz; Instituto de Tecnologia em Fármacos (Farmanguinhos), Rio de Janeiro – RJ, Brazil; eduardo.sousa@fiocruz.br; thiago.frances@fiocruz.br
[3] Universidade Federal do Rio de Janeiro, Instituto de Pesquisa de Produtos Naturais, CCS, Rio de Janeiro – RJ, Brazil.
[4] Universidade Federal de Juiz de Fora, Departamento de Química, Juiz de Fora – MG, Brazil; alexandre.leitao@ufjf.edu.br
[5] University of São Paulo, São Carlos Institute of Physics, São Carlos- SP, Brazil; oliviacarr@gmail.com; chu@ifsc.usp.br
[6] University of Campinas (UNICAMP), Institute of Physics (IFGW), Department of Applied Physics, "Gleb Wataghin," Campinas- SP, Brazil; fmshimizu@yahoo.com.br
* Correspondence: rsangil@iq.ufrj.br (R.A.S. San Gil, Tel.:+55-21-3938-7737)

Figure S1 ^{13}C CPMAS NMR spectra of mefloquine hydrochloride obtained at 9800 and 10000 Hz spinning frequencies. (*) denotes spinning sidebands.

Figure S2 ^{13}C CPMAS NMR spectra of mefloquine hydrochloride (sample MQ), with contact tin 1100µs and a variation of recycle time (s).

Figure S3 ^{13}C CPMAS NMR spectra of mefloquine hydrochloride (sample MQ), with a variation in contact time (in µs).

Figure S4 ^{13}C CPMAS NMR spectra of potassium polacrilin resin (sample R), obtained with a variation in contact time (in µs).

Figure S5 ^{13}C CPMAS NMR spectrum of the mefloquine-resinate (sample MQ-R), with a variation in contact time (in µs).

Figure S6 ^{13}C solution NMR spectrum (DMSO-d_6) of sample MQ.

Figure S7 Distribution of T_{CH} for the carbons of mefloquine, MQ (blue); MQ-R complex (orange); and resin R (green).

Figure S8 Dependence of C2 signal intensity (vertical scale, arbitrary unities) on the contact time t_c (horizontal scale, in s unities) for the samples mefloquine hydrochloride (MQ) and mefloquine-polacrilin complex (MQ-R).

Figure S9 Dependence of C7 signal intensity (vertical scale, arbitrary unities) on the contact time t_c (horizontal scale, in s unities) for the samples mefloquine hydrochloride (MQ) and mefloquine-polacrilin complex (MQ-R).

Figure S10 Dependence of C5 signal intensity (vertical scale, arbitrary unities) on the contact time t_c (horizontal scale, in s unities) for the samples mefloquine hydrochloride (MQ) and mefloquine-polacrilin complex (MQ-R).

Figure S11 Dependence of C6 signal intensity (vertical scale, arbitrary unities) on the contact time t_c (horizontal scale, in s unities) for the samples mefloquine hydrochloride (MQ) and mefloquine-polacrilin complex (MQ-R).

Figure S12 Dependence of C12 signal intensity (vertical scale, arbitrary unities) on the contact time t_c (horizontal scale, in s unities) for the samples mefloquine hydrochloride (MQ) and mefloquine-polacrilin complex (MQ-R).

Figure S13 Dependence of C signal intensity (vertical scale, arbitrary unities) on the contact time t_c (horizontal scale, in s unities) for carbons in the sample of polacrilin resin (R).

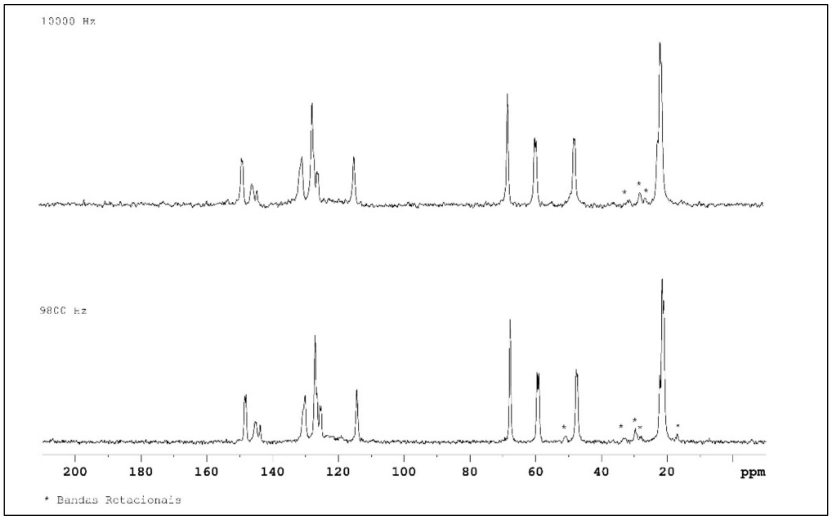

Figure S1 - ^{13}C CPMAS NMR spectra of mefloquine hydrochloride (sample MQ) obtained at 9800 and 10000 Hz spinning frequencies. (*) denotes spinning sidebands.

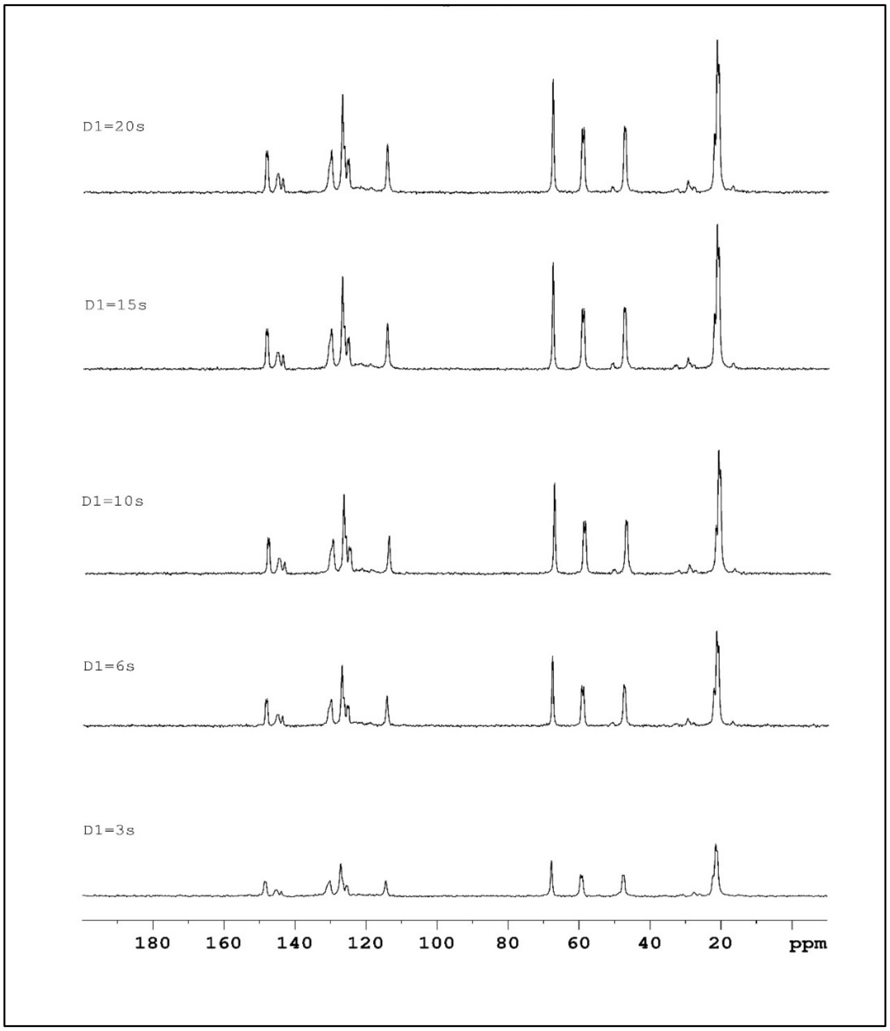

Figure S2 – ^{13}C CPMAS NMR spectra of mefloquine hydrochloride (sample MQ), with contact time 1100µs and a variation of recycle time (s).

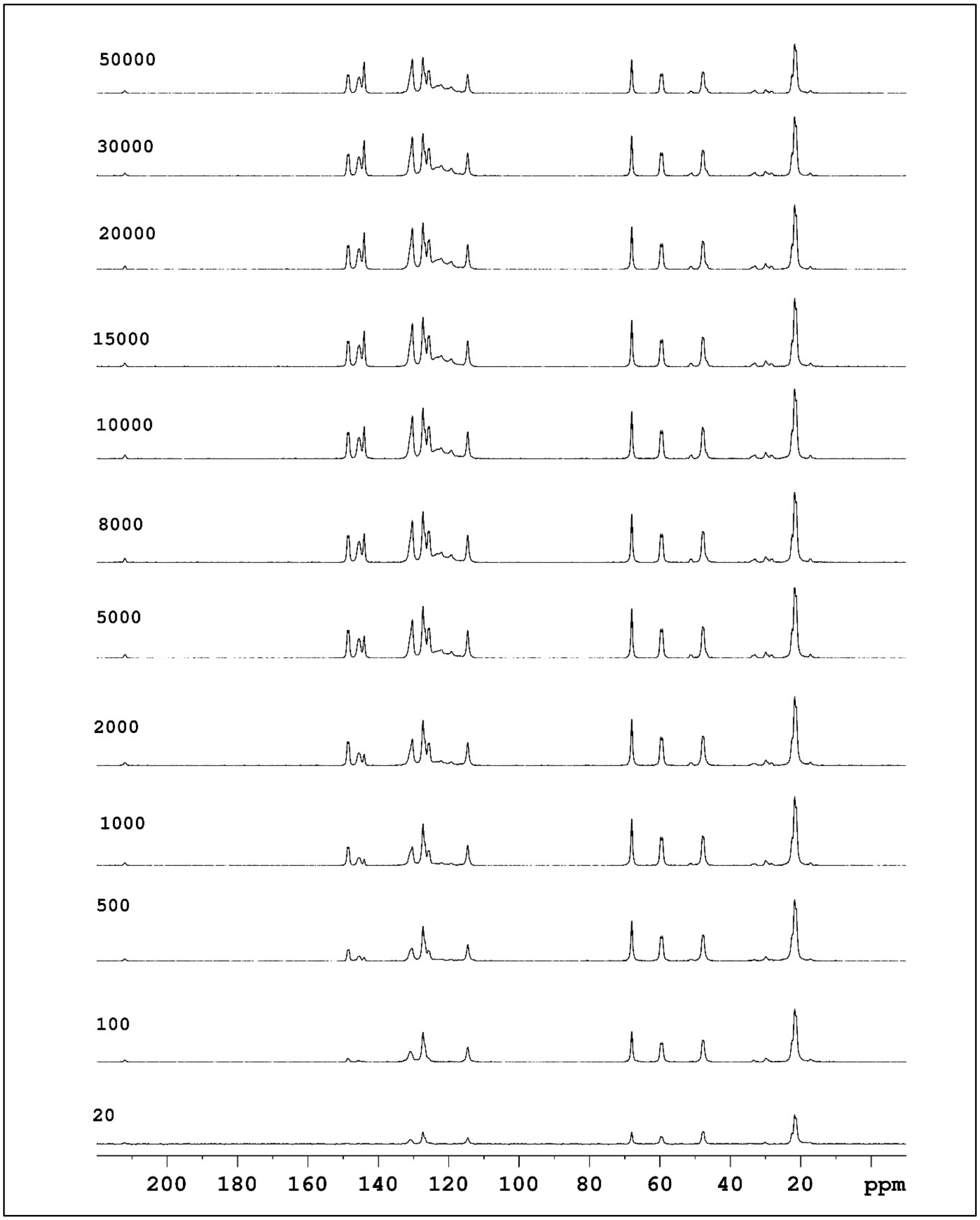

Figure S3 – ^{13}C CPMAS NMR spectra of mefloquine hydrochloride (sample MQ), with a variation in contact time (in µs).

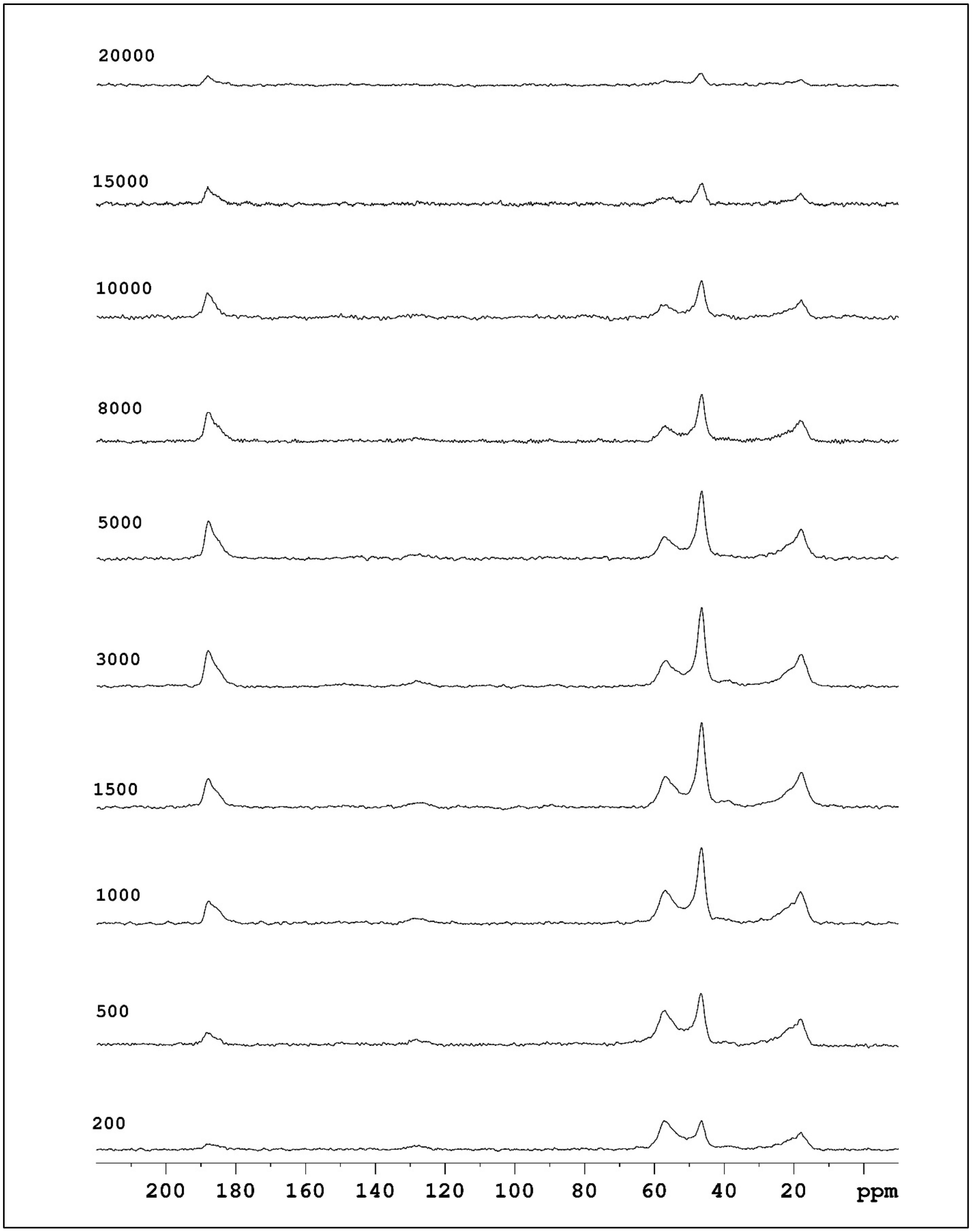

Figure S4 – ^{13}C CPMAS NMR spectra of potassium polacrilin resin (sample R), obtained with a variation in contact time (in µs).

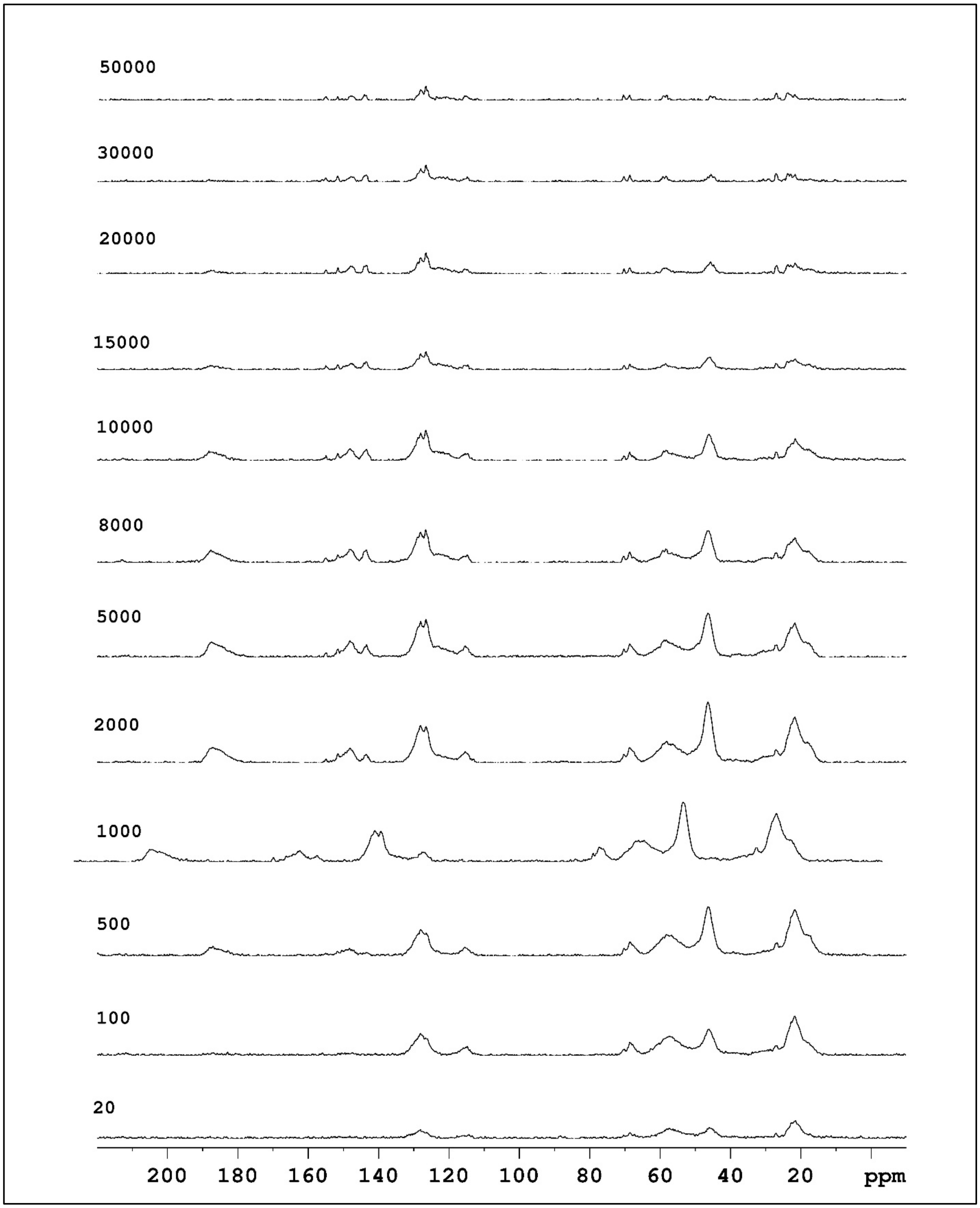

Figure S5 – ^{13}C CPMAS NMR spectrum of the mefloquine-resinate (sample MQ-R), with a variation in contact time (in µs).

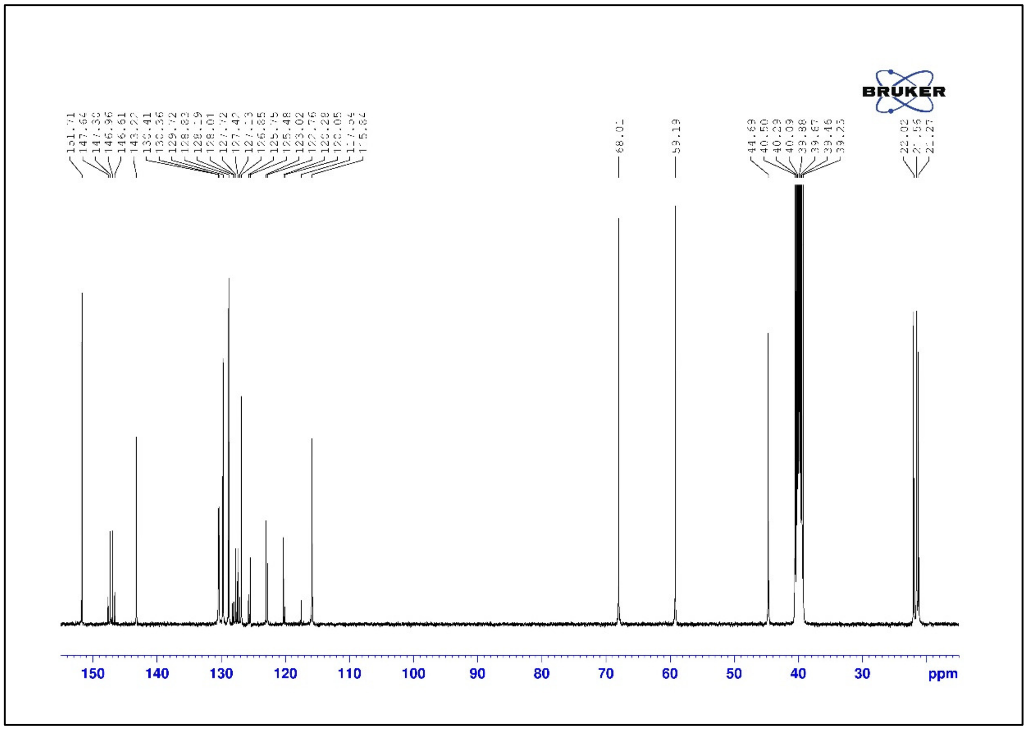

Figure S6 – ^{13}C NMR (500 MHz, DMSO-*d*$_6$) spectrum of the MQ sample.

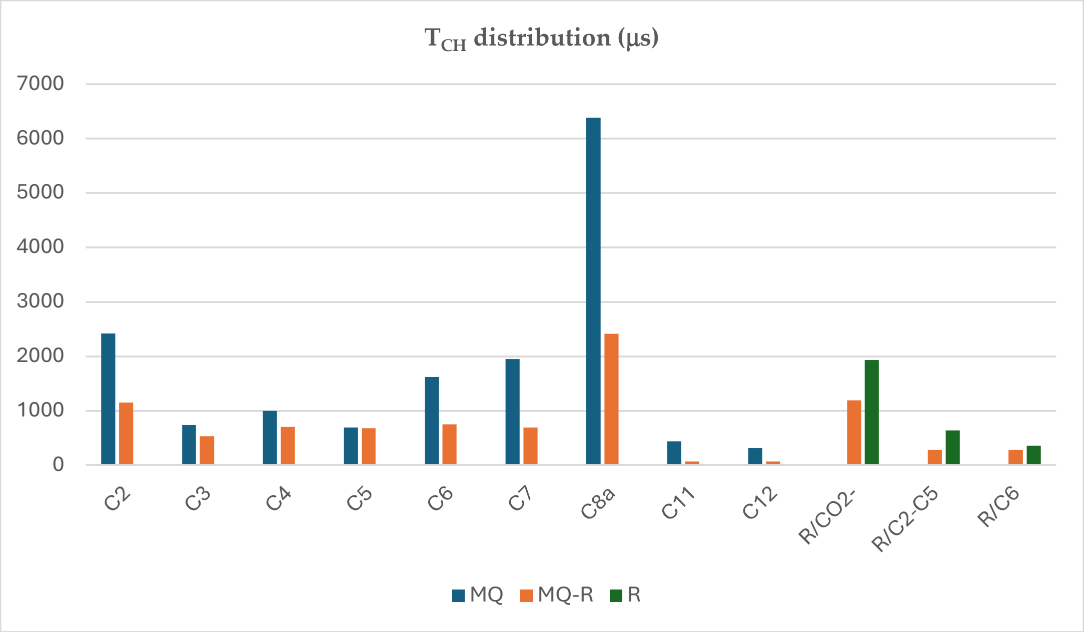

Figure S7. Distribution of T_{CH} for the carbons of mefloquine, MQ (blue); MQ-R complex (orange); and resin R (green).

<u>C2 carbon</u>

Mefloquine

δ = 145.2 ppm

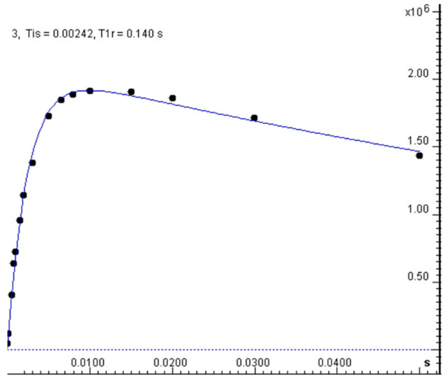

Mefloquine-polacrilin

δ = 148.0 ppm

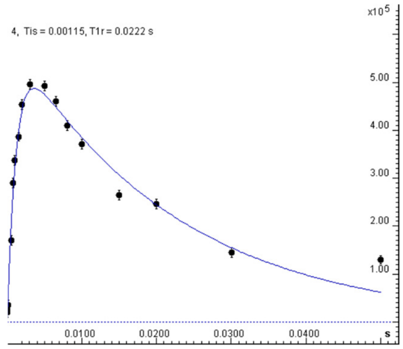

δ = 147.3 ppm

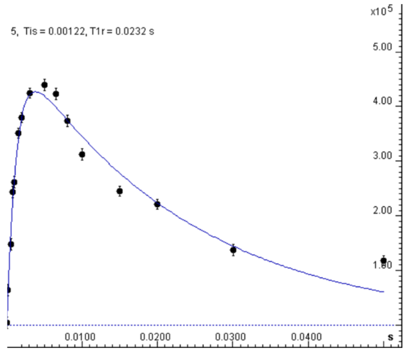

Figure S8. Dependence of C2 signal intensity (vertical scale, arbitrary unities) on the contact time t_c (horizontal scale, in s unities) for the samples mefloquine hydrochloride (MQ) and mefloquine-polacrilin complex (MQ-R).

C7 carbon

Mefloquine | **Mefloquine-polacrilin**

δ = 130.1 ppm | **δ = 128.8 ppm**

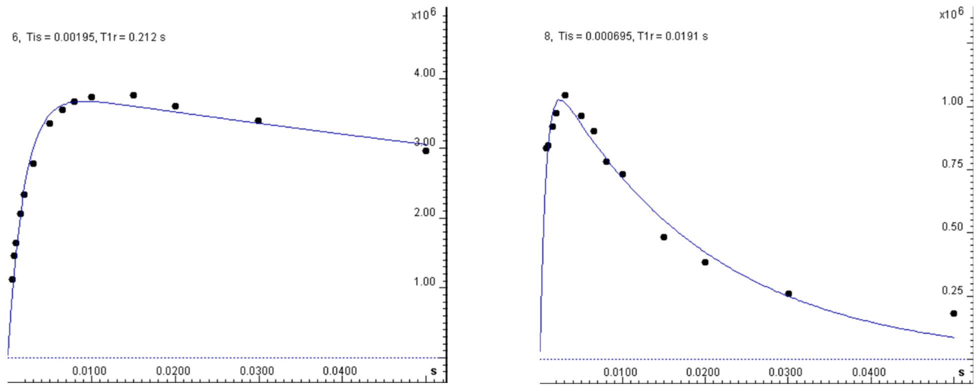

Figure S9. Dependence of C7 signal intensity (vertical scale, arbitrary unities) on the contact time t_c (horizontal scale, in s unities) for the samples mefloquine hydrochloride (MQ) and mefloquine-polacrilin complex (MQ-R).

<u>C5 carbon</u>

Mefloquine

δ = 127.1 ppm

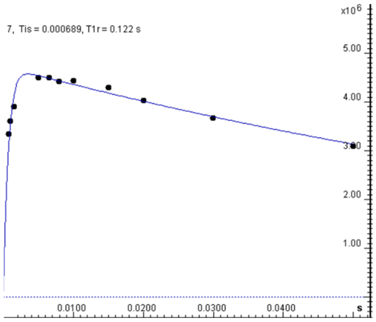

δ = 126.5 ppm

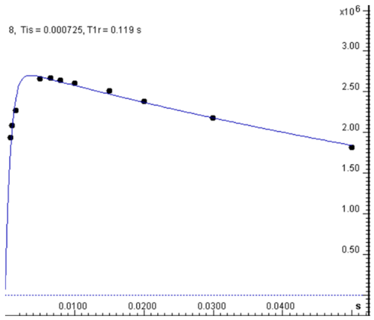

Mefloquine-polacrilin

δ = 128.0 ppm

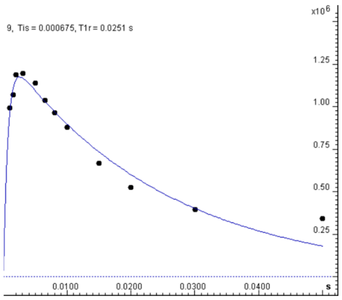

δ = 127.5 ppm

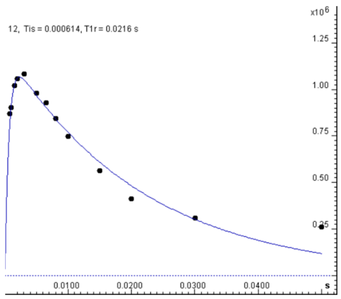

Figure S10. Dependence of C5 signal intensity (vertical scale, arbitrary unities) on the contact time t_c (horizontal scale, in s unities) for the samples mefloquine hydrochloride (MQ) and mefloquine-polacrilin complex (MQ-R).

C6 carbon

Mefloquine

δ = 125.6 ppm

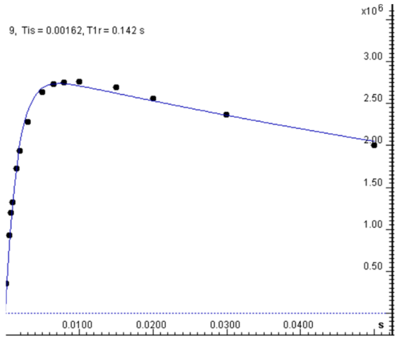

Mefloquine-polacrilin

δ = 126.6 ppm

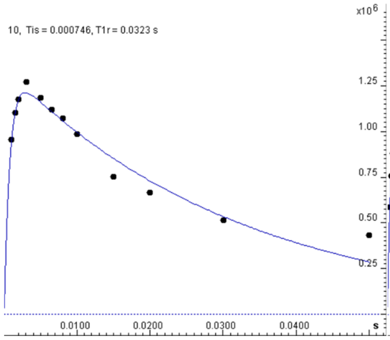

δ = 126.0 ppm

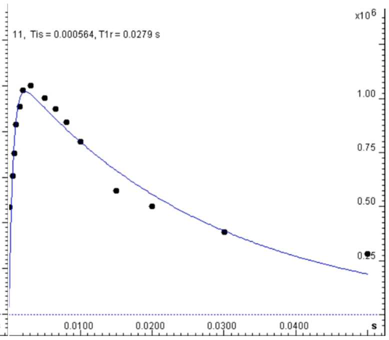

Figure S11. Dependence of C6 signal intensity (vertical scale, arbitrary unities) on the contact time t_c (horizontal scale, in s unities) for the samples mefloquine hydrochloride (MQ) and mefloquine-polacrilin complex (MQ-R).

C12 carbon

Mefloquine

δ = 59.5 ppm

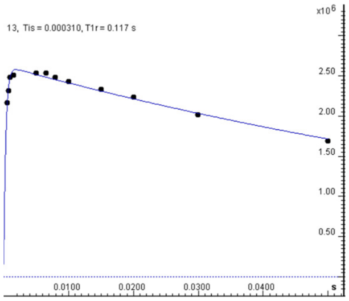

δ = 59.0 ppm

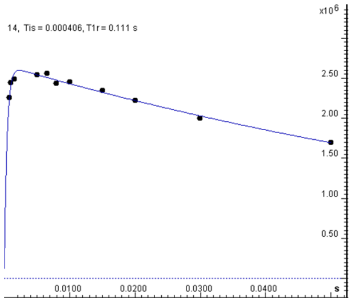

Mefloquine-polacrilin

δ = 58.1 ppm

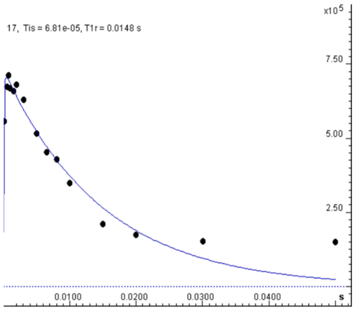

Figure S12. Dependence of C12 signal intensity (vertical scale, arbitrary unities) on the contact time t_c (horizontal scale, in s unities) for the samples mefloquine hydrochloride (MQ) and mefloquine-polacrilin complex (MQ-R).

Polacrilin Resin

C1 (COO^-), δ = 186.5 ppm

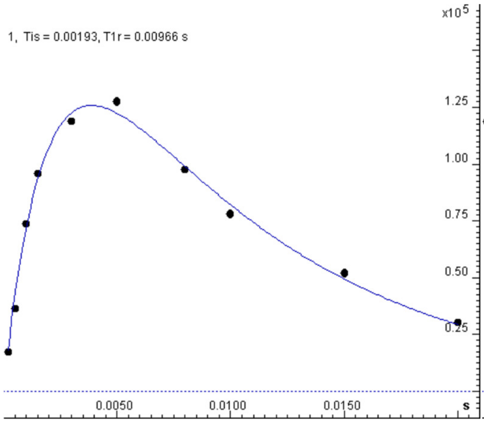

C2, C3, C4, C5, δ = 56.9 ppm

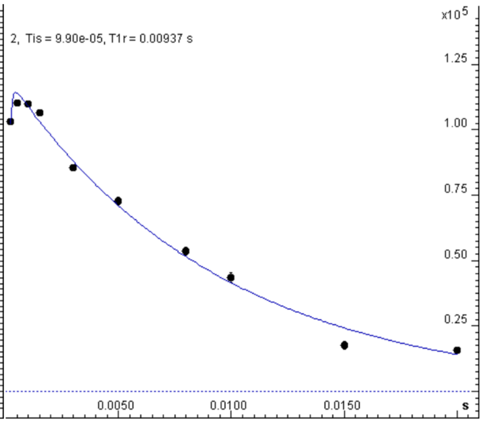

C2, C3, C4, C5, δ = 46.4 ppm

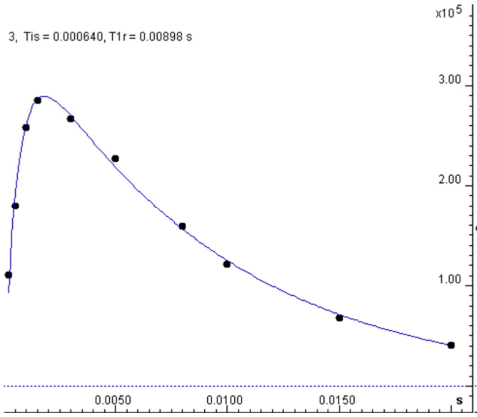

C6 (CH_3), δ = 18.1 ppm

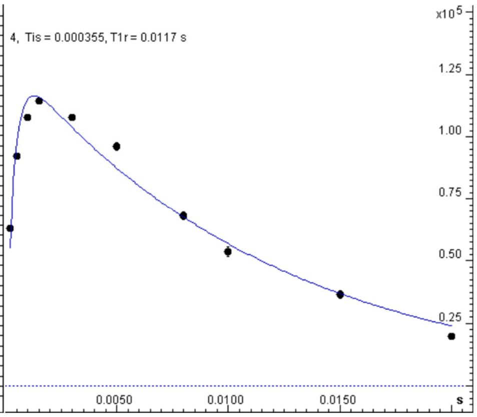

Figure S13. Dependence of C signal intensity (vertical scale, arbitrary unities) on the contact time t_c (horizontal scale, in s unities) for carbons in the sample of polacrilin resin (R).
